# Concurrent enhancement in fire retardancy and mechanical properties of flax/vinyl ester bio-composites via magnesium hydroxide incorporation

**DOI:** 10.1371/journal.pone.0319421

**Published:** 2025-03-11

**Authors:** Rizwan Ahmed Malik

**Affiliations:** Department of Mechanical Engineering, College of Engineering, Prince Sattam bin Abdulaziz University, Al-Kharj, 11942, Saudi Arabia; National Chung Cheng University, Taiwan & Australian Center for Sustainable Development Research and Innovation (ACSDRI), AUSTRALIA

## Abstract

The fire-retardant properties of bio-composites are generally enhanced through nano fillers incorporation at the cost of their mechanical properties. In this study, magnesium hydroxide (MH) nano filler was incorporated into flax/vinyl ester (VE) bio-composite to enhance its fire-retardancy and thermal stability simultaneously with mechanical properties. MH is chemically compatible with cellulosic fibers which played a role in improving the interfacial bonding and hence the mechanical properties in this study. The composites fabrication process parameters including curing temperature and vacuum pressure were also optimized in this study. The concentration of MH was varied as 0, 5, and 10% in the flax/VE composite. The tensile and flexural strengths of the 5% MH filled flax/VE composites were observed to increase by 10% and 48% respectively. This enhancement in strength was attributed to the improved interfacial bonding and compatibility of MH with flax fiber, verified through Field Emission Scanning Electron Microscopy (FESEM) and Fourier Transformed Infrared Spectroscopy (FTIR), respectively. The horizontal burning rate of the composites was decreased by 25% after MH incorporation, which was validated through a limiting oxygen index (LOI) test. The results of cone calorimetry highlighted a decrease of 11.73% in the peak values of heat release rate (HRR) which is a sign of enhancement in fire retardancy. The thermogravimetric analysis also discovered an improvement in the thermal stability of the composites. These bio-composites with improved mechanical, thermal and fire-retardant properties may find their applications in automobiles, marine and aerospace industries.

## 1. Introduction

Natural fiber reinforced composites (NFRCs) are well known replacements of the conventional synthetic fibers for their eco-friendly characteristics [1,2]. Flax fiber is easily available with acceptable mechanical properties and low cost. It is used in a variety of applications including interior parts of automobiles, aerospace, marine, construction, and furniture [3,4]. Besides environmentally friendly characteristics, natural fibers-based composites have poor flammability properties [5]. Cellulose, hemicellulose, and lignin are the major components of a bio-fiber [6]. At high temperatures, these components start degrading which influences the performance of NFRCs [7]. The flammability and fire retardancy mechanism of natural fibers reinforced composites need to be explored in detail. Several researchers have reported improvement in the fire-retardant properties of the natural fiber composites, but as a result, decrease in the mechanical properties occurs due to the incompatibility of the fire-retardant fillers, natural fibers, and polymer matrices [8–11]. Tang et al incorporated intumescent flame retardant in polylactic acid composite which enhanced its thermal properties and fire resistance but reduced the mechanical strength [12]. In another study, double layer hydroxide was incorporated in polypropylene to improve its thermal stability and fire-retardant properties but sacrificed the mechanical properties [13]. Verdolotti et al used ammonium polyphosphate as a flame-retardant filler in a bio-composite which reduced the mechanical properties [14]. The reduction of mechanical properties after addition of flame-retardant fillers in polymer composites is a common reported problem. This paper mainly focuses on improvement in the fire-retardant properties, thermal stability as well as mechanical properties of flax/VE composites concurrently; the possible mechanism has been discussed with reasons.

Different categories of flame retardants exist to enhance fire retardant and thermal properties of polymer composites. Some common classes of flam-retardant fillers include halogens, phosphorous, nitrogen, silicon, carbon, sulphates, clays, and bio-based fillers [15–16]. Some of these fillers have gained significant importance due to their excellent flame-retardant properties, but there are environmental concerns on the use of such fillers [17–19]. On the other hand, hydroxide-based flame-retardant fillers have been discovered as non-toxic materials with no harmful effect on the environment when burnt [20–22]. Magnesium hydroxide (MH) is a common fire-retardant filler from this group which produces excellent fire-retardant effect as well as absorbing toxic gases during combustion of polymers [23–24].

In this study, MH was incorporated into flax/VE composite as a fire-retardant filler. The composites were fabricated using VARTM process. The light weight and acceptable mechanical properties of flax/VE composite make it a suitable choice for application in interior parts of automobiles, aerospace, and marine industry. The concentration of MH filler varied from 0 ~ 10 wt% in this study. Flax/VE specimens were manufactured with and without MH filler and different characterizations were performed to see the influence of MH on the performance of flax/VE composite. When burned, the MH yields into water and magnesium oxide [25]. This decomposition mechanism works in fire retardancy. MgO acts as a char layer over the specimen to protect rapid heat flow during combustion process, while water is a famous fire extinguisher. FTIR spectroscopy of the composites was conducted which discovered an interaction between MH and flax fibers. This interaction caused an enhancement in the mechanical properties of the MH filled flax/VE composites. Tensile and bending strengths were enhanced by 10% and 48% respectively. This interaction between MH and flax fibers was also seen through FESEM (Field Emission Scanning Electron Microscope) study, the MH particles were seen deposed on the surfaces of flax fiber. Improved interfaces were observed in the case of MH filled flax/VE composites. To evaluate the influence of MH on flammability and thermal stability of flax/VE composites, a horizontal flame test, LOI test, cone calorimetry, and thermogravimetric analysis were performed. Fire retardancy and thermal stability of the composite were enhanced after MH addition.

The novelty of this study exists in the concurrent enhancement of the mechanical properties as well as fire resistance and thermal stability of the bio-composite. Generally, the incorporation of fire-retardant fillers enhances fire resistance but causes to decrease the mechanical strength of the composites which has been addressed in this work through incorporation of a chemically compatible fire retardant filler in the bio-composites.

## 2. Materials

Flax fabric (Bi-directional:150 g/m^2^, Density: 1.4 g/cm^3^, Tensile strength: 250–300 MPa) was obtained from LINEO ITS Composites (China). Vinyl ester resin (specific density =  1.02, & viscosity =  150 cps) and its hardener (Butanox M-60) were purchased from CCP Composites (Korea). Magnesium hydroxide powder (mol. weight =  58.32 g/mol, particle size <  100 nm) was purchased from Sigma-Aldrich (USA).

## 3. Composites fabrication

**Fig 1:**
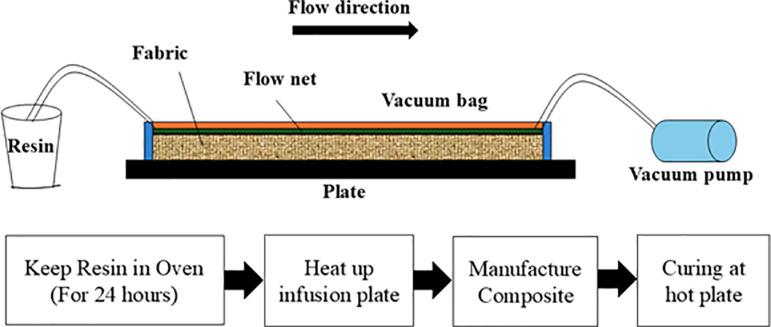
Fabrication process of the composites.

All the flax/VE and MH/flax/VE compositions were fabricated using VARTM method. The fiber volume fraction was 40%. The composites fabrication method has been explained in Fig 1. Before fabrication, the vinyl ester resin was kept in an oven at 30 °C for 24 hours to keep viscosity as provided by the supplier. The flax fabric (8 layers) was stacked up on the pre-heated clean metallic surface and covered with a vacuum bag. The setup was connected to a vacuum pump to evacuate the air from the mold. After maintaining the vacuum for 5 minutes, the resin was allowed to flow into the mold passing through the piled up flax fabric utilizing the vacuum pressure and a flow net. The mold was kept on a hot autoclave surface for curing the composites at high temperature. As the curing temperature and vacuum pressure play an important role in composites fabrication, these parameters were optimized as follows.

### 3.1. Resin flow

The flow of VE resin through the flax fabric can be represented by the Darcy’s law as:


$$dq=\left[\frac{\partial }{\partial x}\left(\frac{k_{xx}}{\mu }\frac{\partial p}{\partial x}\right)+\frac{\partial }{\partial y}\left(\frac{kyy}{\mu }\frac{\partial p}{\partial y}\right)+\frac{\partial }{\partial z}\left(\frac{kzz}{\mu }\frac{\partial p}{\partial z}\right)\right]dxdydz$$ (1)

In equation 1, p denotes the vacuum pressure during the VARTM process, μ represents the viscosity of VE resin, while *k*_*ij*_ symbolizes the permeability of the flax fabric [26]. According to equation (1), the resin flow (q) is promotional to vacuum pressure (∂p), and the inverse of viscosity (1/μ).

**Fig 2:**
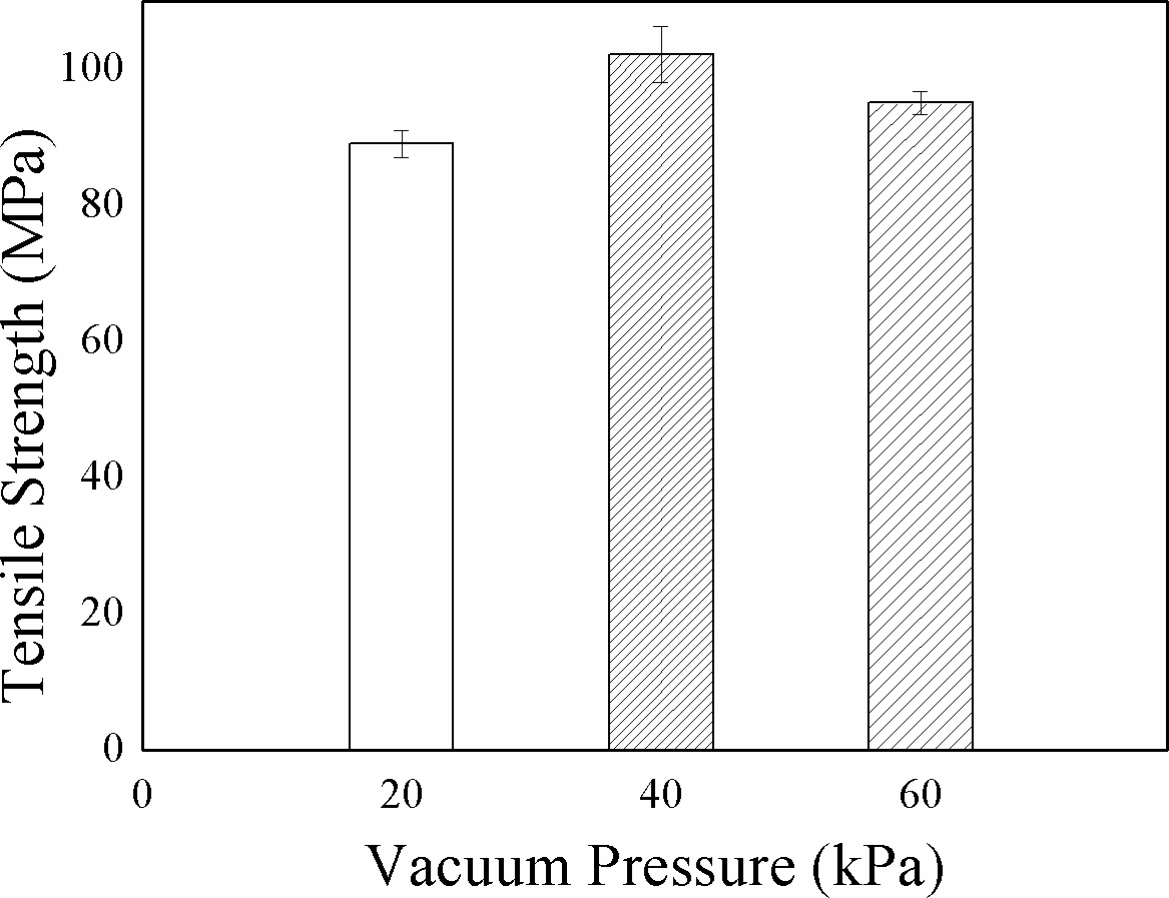
Influence of vacuum pressure on tensile strength of the composites.

To determine the influence of pressure on the composite properties, the flax/VE composites were prepared under varying vacuum pressures of 20 kPa, 40 kPa and 60 kPa. To keep the viscosity of the VE resin uniform, it was kept at 30 °C in an oven for one hour before the composites’ fabrication. However, curing was allowed at room temperature. Results of the tensile test under varying vacuum pressure are shown in Fig 2. As predicted above, the vacuum pressure influences the properties of the composites. The results show that the flax/VE composite with highest tensile strength (102.2 MPa) was fabricated at 40 kPa vacuum pressure, which has been considered as an optimized vacuum pressure for the remaining study.

### 3.2. Resin curing

Viscosity is an important parameter of thermosetting resins during resin transfer molding processes. It depends upon multiple factors as shown below [27].


μ=μ(T,γ,β) (2)

**Fig 3:**
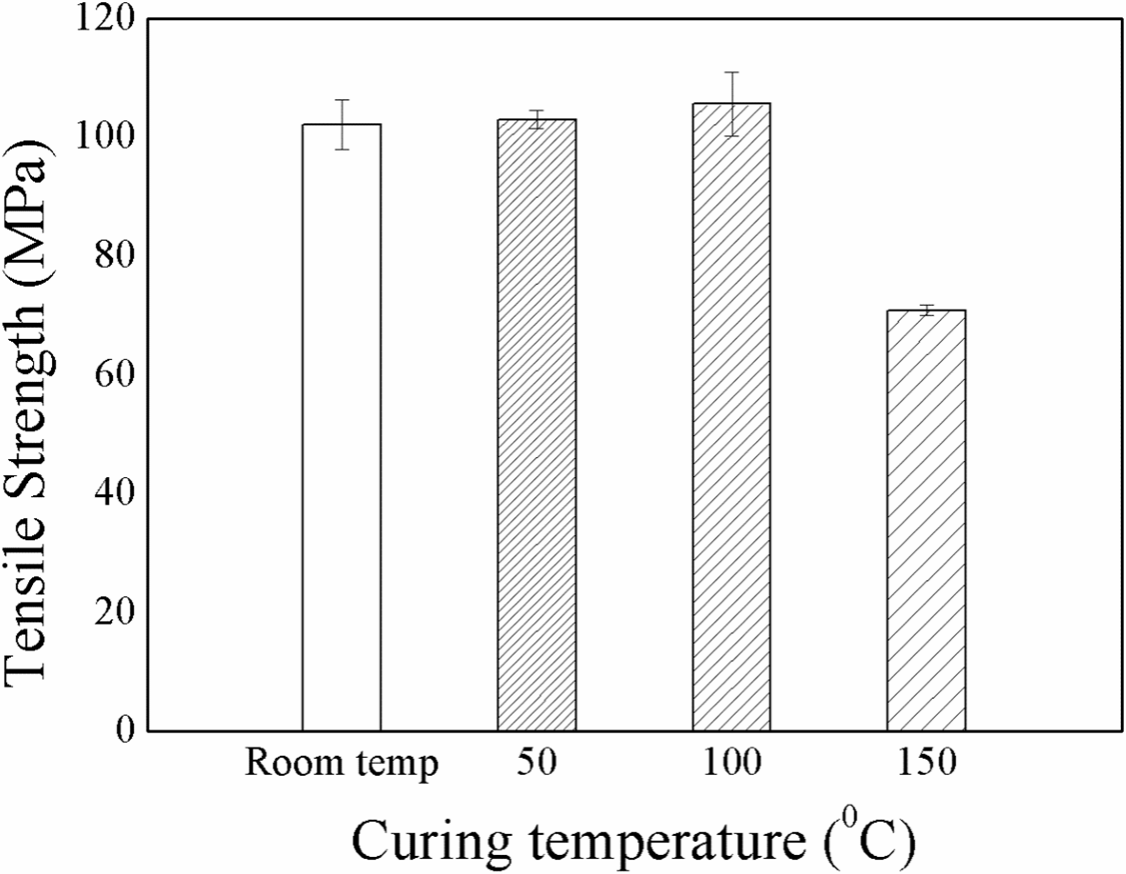
Influence of curing temperature on tensile strength of the composites.

In equation 2, T is curing temperature, γ is shear rate, and β represents the isothermal degree of cure. The influence of curing temperature on the properties of the composites was also evaluated in this study. The curing temperature varied from room temperature to 50 °C, 100 °C and 150 °C while the vacuum pressure was 40 kPa (as optimized previously). After curing for 5 hours, specimens were cooled at room temperature. Influence of the curing temperature over the tensile strength of flax/VE composite is shown in Fig 3. Flax/VE composites fabricated at 100 °C curing temperature had the highest tensile strength among all composites. Curing beyond 100 °C resulted in a lower tensile strength.

### 3.3. Fabrication of MH filled flax/VE composites

The remaining composites were prepared with the optimized parameters of VARTM (100 °C curing temperature and 40 kPa vacuum pressure). Three different types of compositions were prepared for further study, which are given in Table 1. Each composition is made of 8 layers (630 mm x 380 mm) of woven flax fabric. To remove any moisture from the flax fabric before fabrication of the composites, it was initially kept in oven for 5 hours at 100 °C. Hardener and accelerator were stirred into the VE resin for 10 minutes at 600 RPM. MH powder was equally spread between each layer of flax fabric using hand layup method. The composite was allowed to be cured at 100 °C on a hot plate for 5 hours, and then at room temperature for the next 10 hours. The process sequence was the same as explained in Fig 1.

**Table 1:** Composition and nomenclature of the composites.

Nomenclature	Composite	Weight % of MH
Flax/VE	Flax/Vinyl Ester	0
5M-flax/VE	MH/Flax/Vinyl Ester	5
10M-flax/VE	MH/Flax/Vinyl Ester	10

## 4. Characterizations

### 4.1. FTIR spectroscopy

The FTIR spectroscopy was performed to discover any possible interaction of MH with the flax fibers. JASCO FT-IR-6300 spectrometer (UK) was employed for this study. Transmittance spectra (4000 – 400 cm^−1^) were recorded with 32 scans in each case at a resolution of 4 cm^−1^. The potassium bromide (KBr) disk method was used for this analysis.

### 4.2. Flame test

The flammability of the composite specimens was evaluated through a horizontal flame test. The test was carried out according to ASTM D635 standard. Burning properties of the 75 mm long and 13 mm wide specimens were calculated.

### 4.3. Limiting oxygen index (LOI) test

The ASTM D2893 standard was followed to measure the percentage values of LOI for all the composites using an oxygen index instrument (FESTEC International Co. Ltd., Korea).

### 4.4. Cone calorimetry

A cone calorimeter of Fire Testing Technology (UK) was used to calculate the heat release rate (HRR) of the composites as a function of temperature as per ASTM E1354 standard. Samples of (3 ×  100 ×  100) mm^3^ were tested at a heat flux of 50 kW/m^2^.

### 4.5. Thermal analyses

Thermogravimetric analysis (TGA) and derivative thermogravimetry (DTG) of the composite specimens were performed between room temperature and 800 °C at a heating rate of 10 °C/min in N_2_ inert environment. SDT Q600 thermal analyzer was used for this purpose.

### 4.6. Tensile test

The tensile test was performed at a head speed of 2 mm/min using MTS 97 kN load cell following the ASTM D3039 standard in room conditions. The strain was measured using an extensometer. Five specimens of each composite were tested, the numerical values of strength and modulus in this study are average values while the error is represented by the standard deviation. Tensile test specimens were cut from composite sheets using a vertical saw cutter, each specimen was 25 mm wide and 250 mm long, while gage length was 138 mm. Glass tabs were attached to the ends of the specimens for better holding in the machine jaws; each tab length was 56 mm.

### 4.7. Flexural test

The three point bending test was carried out using a 5 tons Unitech load cell with a 2 mm/min head speed at room conditions. The experiment followed ASTM D790 specifications. The numerical values and errors were calculated as explained in the tensile test section. Three points bending test specimens were cut from the composite sheets such that each specimen was 13 mm wide, while the span length was kept as 16 times the thickness of each specimen.

### 4.8. FESEM analysis

FESEM analysis of the tensile and bending fractured surfaces was carried out using a high-resolution field emission scanning electron microscope (model: Zeiss Gemini (Germany)). To enhance the image quality, the surfaces were first sputtered with gold coating to mitigate the effect of charging. Images were captured at multiple resolutions (500x and 1500x). This study was conducted to observe the interfaces before and after MH filler incorporation in flax/VE composites.

## 5. Results and Discussion

### 5.1. Fourier transformed infrared spectroscopy

**Fig 4:**
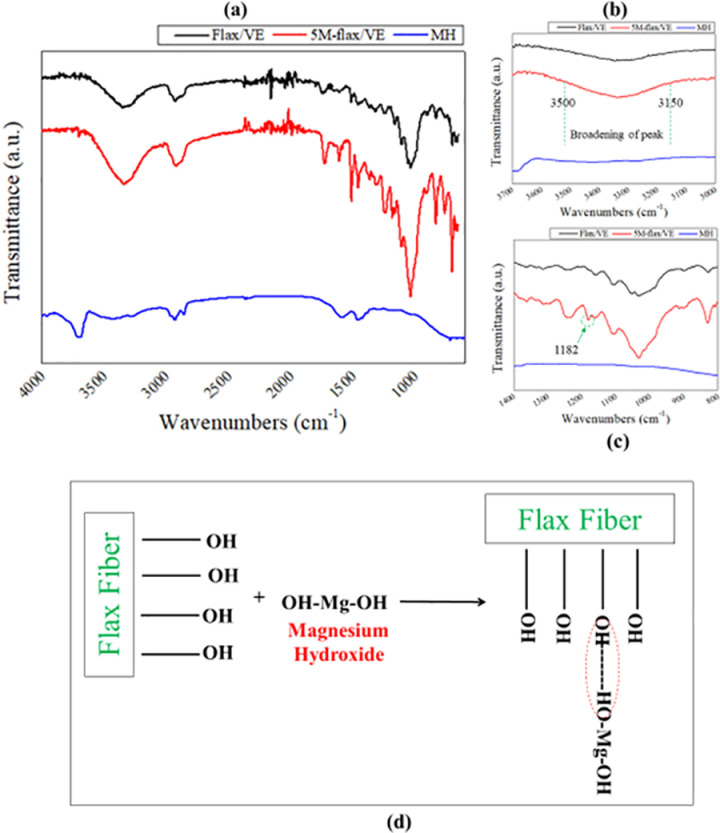
FTIR spectra of the composite in (a) 4000 – 500 range (b) 3700 – 3000 range (c) 1400 – 700 range (d) possible interaction mechanism.

FTIR spectral analysis of MH, flax/VE and 5M-flax/VE composite specimens was conducted to study any possible interaction between MH and flax fibers. The FTIR spectra are shown in Fig 4. The stretching mode of –OH group in MH is represented by a sharp vibration band at 3695 cm^-1^. Residual acetoxy groups are represented by vibration band between 1700 – 1200 cm^−1^, the same has been reported in the literature [26]. The peaks in the spectra of the flax/VE and 5M-flax/VE composites are almost identical except in the fourth region (1400 – 600 cm^−1^). A magnified view of the spectra between 3700 – 3000 cm^−1^ is shown in Fig 4(b). The broadening of the peak between 3500 – 3150 cm^−1^ range for 5M-flax/VE composite indicates an O–H stretch through hydrogen bonding. This has been explained in Fig 4(d) as a possible interaction between the hydroxyl groups in MH and flax fiber. A new peak appeared in the fingerprint region of the spectrum of 5M-flax/VE composite at 1182 cm^−1^, as shown in Fig 4(c), which shows C–N stretch. The presence of interaction between the constituents of the 5M-flax/VE composite encourages the enhancement in tensile strength which will be explained in the tensile properties section.

### 5.2. Flammability

**Fig 5:**
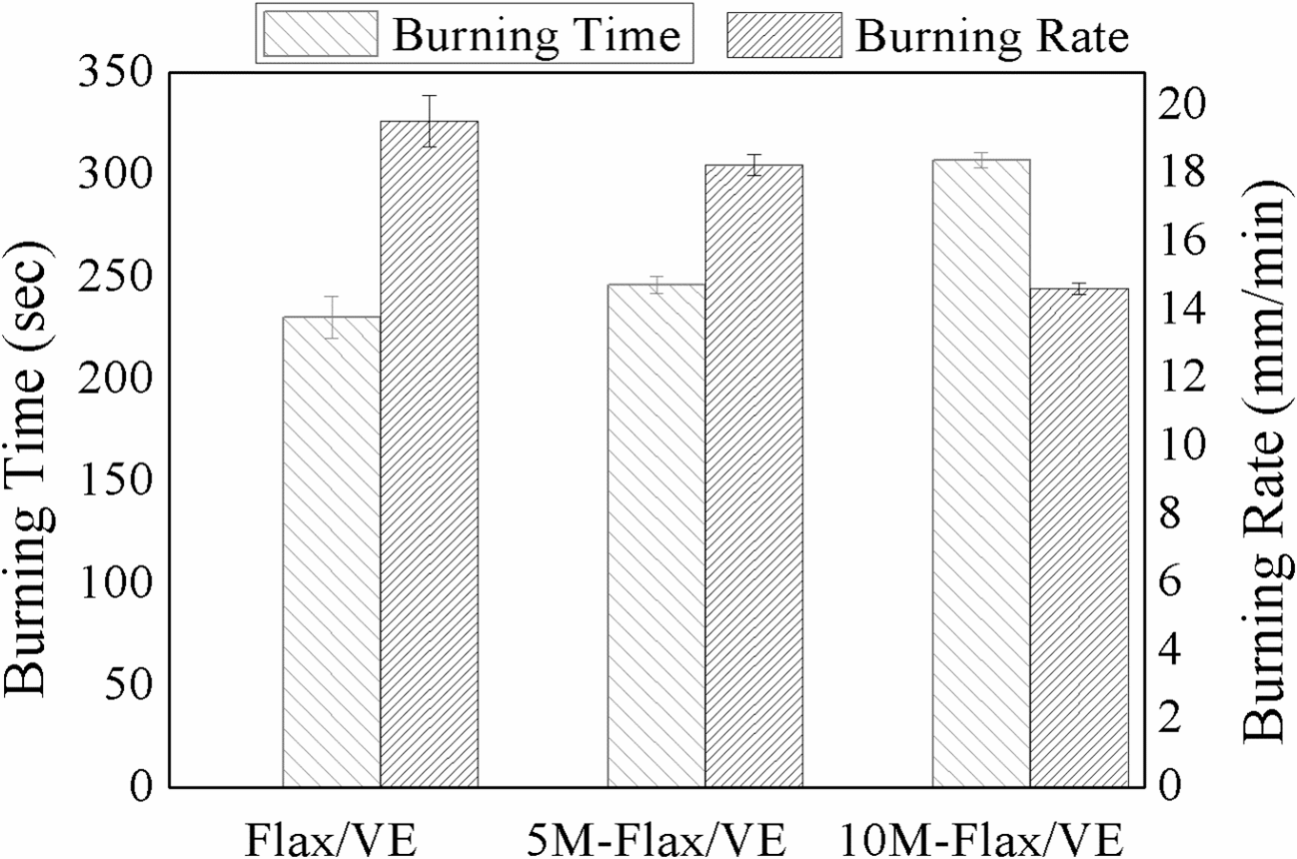
Burning time and burning rate of the composite under horizontal burning.

In the horizontal flame test, the specimens were burnt horizontally. The burning time of each specimen was noted between two reference marks. The distance between two reference marks was 75 mm. Burning rate was calculated from the burning time (t) as 75/t. Fig 5 shows the average burning time and burning rate of each composition. Burning time of the specimens was observed to prolong because of MH incorporation into flax/VE composites. 10M-flax/VE was found to have maximum burning time (307 sec) while flax/VE without MH filler had the shortest burning period (230 sec). The burning rate was observed to decrease by 25% in the case of 10M-flax/VE as compared to flax/VE. On combustion, the magnesium hydroxide decomposes into magnesium oxide and water vapors. The magnesium oxide acts as a thermal barrier on the outer surface of the composites in the form of char layer while the water vapors extinguish the fire. The char layer reduces the propagation of flame by restricting the flow of the heat. Numerical results of the flame test are given in Table 2.

**Table 2:** Fire retardant and thermal properties of all the composites.

Composition	Flame test		LOI	Cone Calorimetry		TGA	
	Burning time (sec)	Burning rate (mm/min)	LOI (%)	pHRR (W/g)	THR (MJ/m^2^)	Onset Temperature (°C)	Residue left (%)
Flax/VE	230	19.56	19.4	402.75	304.21	352	6.93
5M-flax/VE	246	18.27	23.6	375.91	242.94	348	11.36
10M-flax/VE	307	14.66	24.9	353.34	225.33	345	16.69

Alternatively, the results of the flame test have been verified through the LOI test. The LOI of flax/VE composite was observed to the lowest (19.4%). It enhanced to 23.6% and 24.9% for the 5M-flax/VE and 10M-flax/VE composites. The net increase in the LOI of the 10M-flax/VE composite is 28% of that of flax/VE composite which is relatable to the results of the horizontal burning test. The values of the LOI test are shown in Table 2.

**Fig 6:**
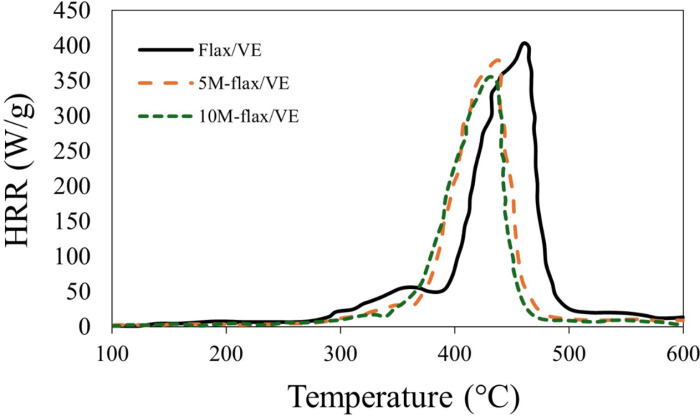
Heat release rate (HRR) of the composites.

**Fig 7:**
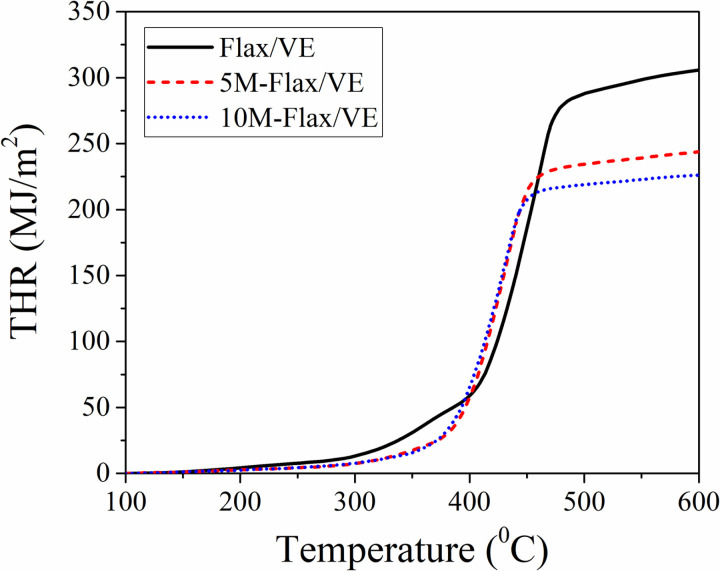
Total heat released (THR) by the composites.

To further explain the flammability phenomenon in the composites, HRR, THR, and pHRR were measured using cone calorimetry, the results are shown in Fig 6, Fig 7 and Table 2, respectively. The results of the cone calorimetry are synchronous with those of LOI and horizontal burning tests. The pHRR of the composites was in the order of flax/VE (402.75 W/g) >  5M-flax/VE (375.91 W/g) >  10M-flax/VE (353.34 W/g). The pHRR was observed to decrease by 11.73% after incorporation of 10% MH in the flax/VE composite, which is a sign of enhancement in fire retardancy. Again, MH releases water vapors and produces a char layer over the composites during combustion which resulted in controlling the release of heat and the HRR was observed to decrease. The THR was also observed to decrease with the incorporation of MH in the flax/VE composite. The values of THR at the end of the test in descending order were recorded as flax/VE (304.21 MJ/m^2^) >  5M-flax/VE (242.94 MJ/m^2^) >  10M-flax/VE (225.33 MJ/m^2^).

### 5.3. Thermal analyses

**Fig 8:**
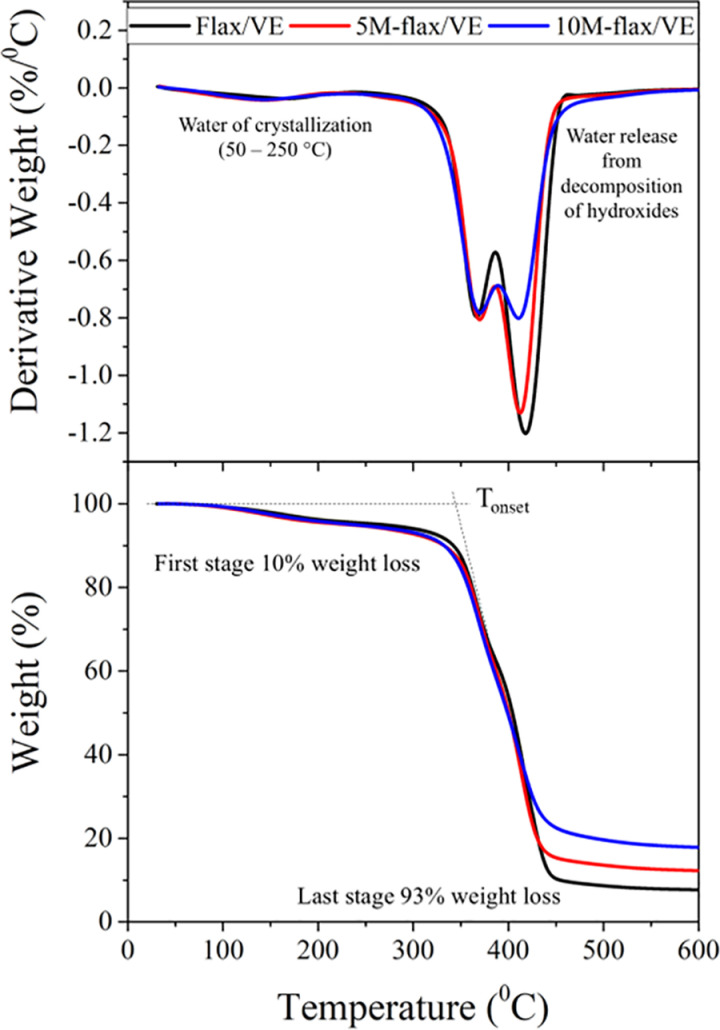
TGA and DTG curves of the composites.

The TGA and DTG thermograms of the composites are shown in Fig 8. The onset of degradation (T_onset_) was calculated from TGA plot by finding a point of intersection between the initial weight baseline and the tangent line to the curve at its maximum gradient. The T_onset_ is the temperature where prominent thermal degradation starts. The curves show a two-stage degradation mechanism for all the composites. In the beginning, a quick fall in the curve denotes the evaporation of moisture. The first major degradation occurred between 300 – 400 °C temperature region, which is visible in the TGA curve as the weight loss occurs in the onset of degradation region, which has been complemented by the DTG curves with a peak around 367 °C. The T_onset_ for flax/VE composite was recorded as 352 °C while it decreased slightly for MH filled composites. At the first stage, there is no prominent difference between the degradation behavior of the MH filled composites. As MH liberates moisture on burning, therefore the onset of degradation for MH filled composites is slightly earlier than that of unfilled flax/VE composite. The second stage of degradation occurred between 400 °C – 450 °C. A steep fall in the weight of the composites can be seen in the TGA curves, which can be identified in the DTG curves at peak between 410 – 425 °C. At the end, the total loss of weight of 10M-flax/VE was observed around 83.1% while the flax/VE lost around 93% its weight. Flax fiber is composed of cellulose, hemicellulose, and lignin. The breakdown of these components of flax fiber during heating is responsible for its weight loss. Magnesium hydroxide decomposes into water vapors and magnesium oxide layer over the composite surface, which acts as a thermal barrier and protects the final residue at the end. When compared with the results of HRR in the cone calorimetry analysis, the T_onset_ in the TGA curve comes in the same temperature region where the HRR curve displayed it first minor peak; however, the last stage of TGA occurs in the same temperature range where the HRR curve displayed its peak value and hence a major weight loss can be seen in the TGA curve.

### 5.4. Tensile properties

**Fig 9:**
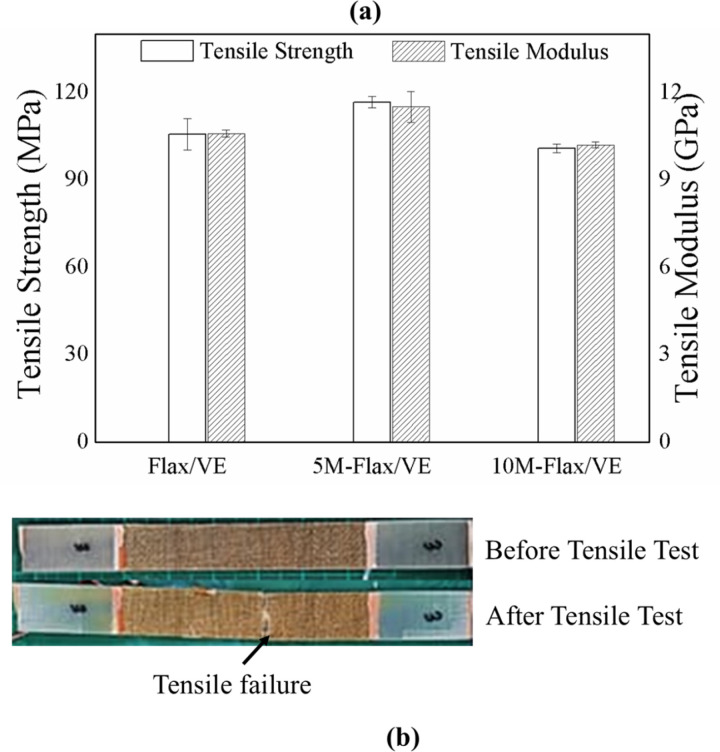
Results of the tensile test (a) tensile strength and modulus (b) specimen before and after tensile test.

A tensile test was performed to study the effect of MH filler on tensile strength and modulus of flax/VE composite. The test results are plotted in Fig 9(a), while a specimen before and after failure is shown in Fig 9(b). The tensile strength and tensile modulus of the Flax/VE composite were determined as 105.7 MPa and 10.6 GPa respectively. An increase in the tensile properties was observed after the incorporation of MH in the composite. 5M-flax/VE composite displayed the highest tensile properties, with tensile strength equal to 116.7 MPa and tensile modulus equal to 11.5 GPa. The increase in tensile strength and tensile modulus was noted as 10% and 9% respectively. The tensile properties were noticed to decrease again after further increasing the concentration of MH filler in the composite (10-flax/VE), which may be attributed to the agglomeration of MH particles causing high stress concentration. In literature, fire retardant particles filled composites are reported to have decreased tensile strength [8]. But in this case, 5M-flax/VE has more tensile strength than the pure flax/VE composite. The reason for the increase in the tensile strength is the possible chemical interaction between MH filler and flax fibers which has been explained in the FTIR analysis. Cellulose is a major part of all natural fibers, the -OH group of cellulosic flax fibers and MH filler makes interaction, which strengthens the composites. The 100 °C curing temperature provides favorable conditions for this O-H bonding to occur. Hence 5M-flax/VE composite is the best option for the applications where tensile properties are of more importance. However, the decrease in tensile properties of 10M-flax/VE is very small, and very near to the flax/VE composite. Tensile properties of all the composite are listed in Table 3.

**Table 3:** Mechanical properties of all the composites.

Composition	Tensile properties		Flexural properties	
	Strength (MPa)	Modulus (GPa)	Strength (MPa)	Modulus (GPa)
Flax/VE	105.6 ± 5.37	10.6 ± 0.12	95.3 ± 13.86	6.28 ± 0.14
5M-flax/VE	116.7 ± 2.00	11.5 ± 0.53	141.7 ± 13.56	7.04 ± 0.16
10M-flax/VE	100.8 ± 1.54	10.2 ± 0.10	123.5 ± 7.37	8.74 ± 0.19

**Fig 10:**
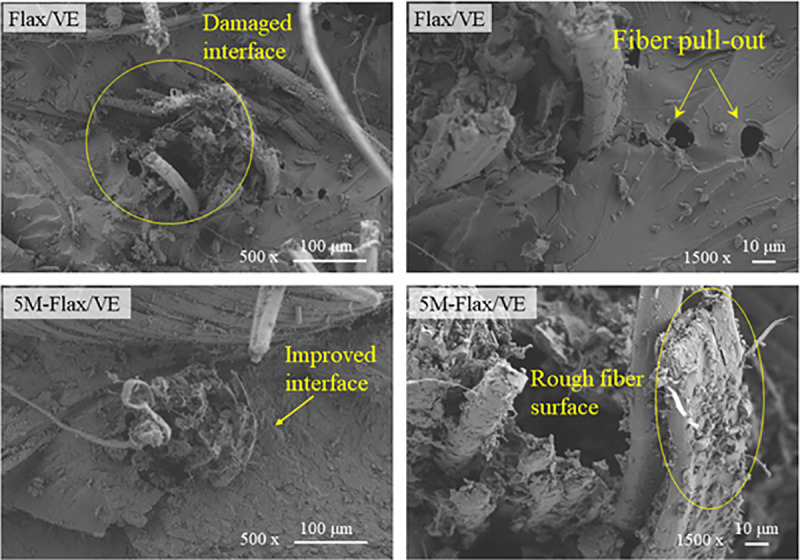
FESEM images of the composite surfaces fractured after tensile test.

Fig 10 shows the FESEM images of flax/VE and 5M-flax/VE specimens after tensile tests. Images of each specimen at two different resolutions (500x and 1.5kx) are presented. The fiber matrix interface can be clearly seen in these images. Fiber-matrix interfacial damage can be seen in the FESEM images of the flax/VE, which is due to possible fiber pull-out failure mode. In the FESEM images of 5M-flax/VE specimen, MH particles can be seen attached to the surface of the fibers, which made the fibers surfaces rough. The rough fiber surfaces make stronger physical bonding with the matrix, and offer more resistance during fiber pull-out, and hence no fiber pull-out failure was observed in the FESEM images of 5M-flax/VE. The MH particles were seen bonded to the fibers due to the possible chemical interaction explained above.

### 5.5. Flexural properties

**Fig 11:**
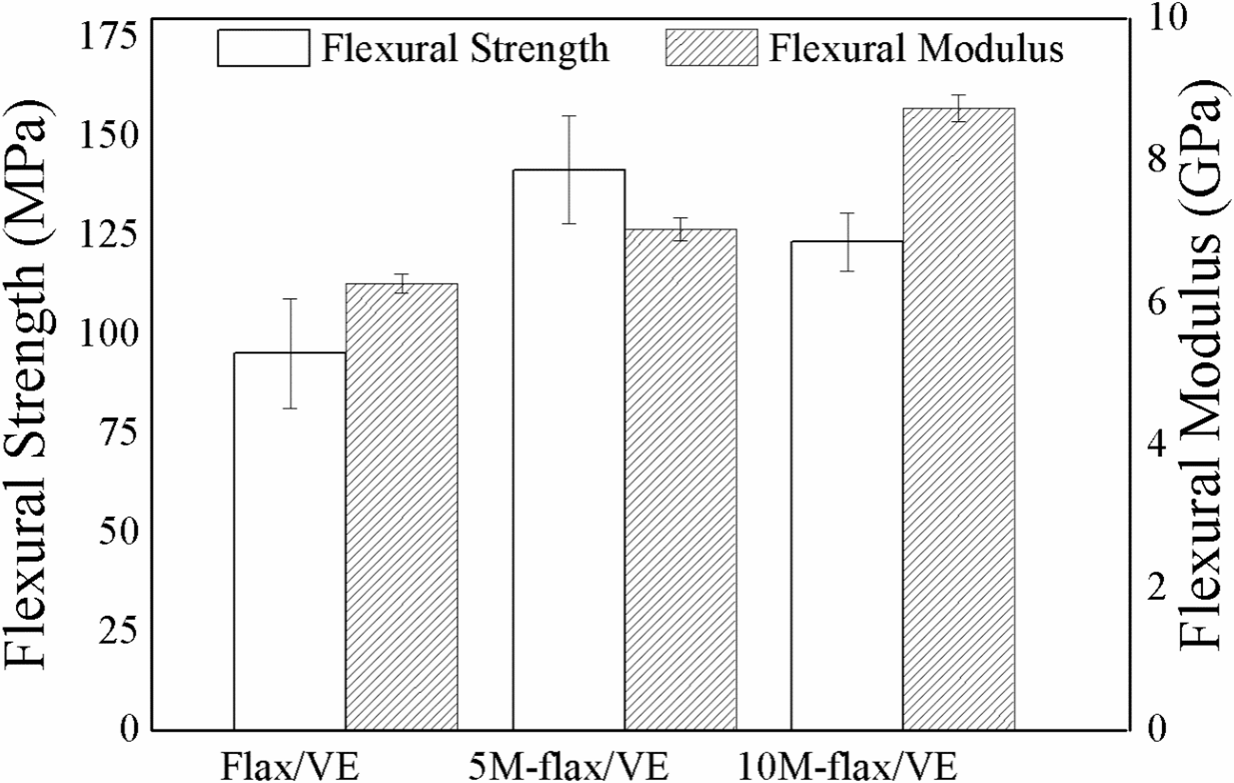
Flexural strength and modulus of the composites.

The flexural properties of each composite are plotted in Fig 11. The flexural strength and flexural modulus of flax/VE composite were recorded as 95.3 MPa and 6.28 GPa respectively. Like tensile properties, the flexural properties were also found to increase after MH addition in flax/VE composite. The 5M-flax/VE composite was found to have maximum flexural strength (141.7 MPa) which is 48% more than the flexural strength of flax/VE composite. The flexural strength of 10M-flax/VE composite was also more than the strength of flax/VE blend but less than that of 5M-flax/VE. The reason for the increase in flexural strength is the same as discussed in the tensile test case. The possible chemical interaction between flax fibers and MH fillers makes the fibers surfaces rough and improves the interfacial bonding. Flexural properties of the composites are presented in Table 3.

**Fig 12:**
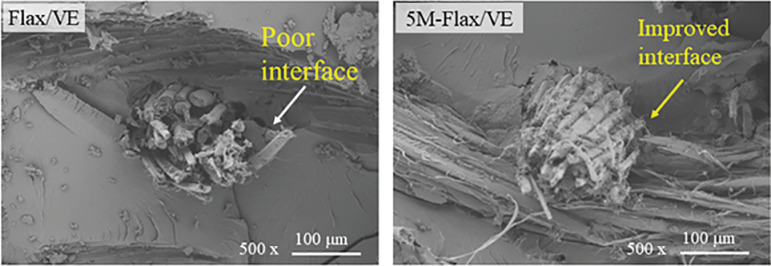
FESEM images of the composite surfaces fractured after flexural test.

Fig 12 shows the FESEM images of flax/VE and 5M-flax/VE composites after bending tests. The fibers-matrix interfacial areas are focused in the micrographs. 5M-flax/VE has improved interfaces as compared to that of flax/VE composite, which is the reason for its higher flexural strength.

## 6. Conclusions

The study was aimed to concurrently enhance the fire retardancy, thermal stability and mechanical properties of flax/VE composite, for which MH nanofiller was incorporated into the composite. MH is chemically compatible to flax fiber due to their O–H interaction as discovered by the FIR analysis during this study. This compatibility caused an enhancement in the mechanical properties of the composites. Additionally, the desired fire-retardant properties and thermal stability were also achieved due to the char formation capability of MH during combustion. The failure mechanism of the composites was verified with the help of FESEM micrographs. The FESEM images revealed that the interfaces between flax fibers and VE resin were enhanced in the presence of MH particles, which changed the composite’s failure behavior from fiber pull-out to fiber breakage failure. The 5 wt% MH incorporation was observed to give improved fire retardant, thermal and mechanical properties. The burning rate was found to decline by 25%, while the total heat released was reduced by 12.12%. Also, the tensile and flexural strength increased by 10% and 48% respectively. Due to their light weight and improved properties these composites can be used in manufacturing the interior parts of automobiles, aerospace and marine. However, the concentration of MH filler can be decided based on specific application. The future study may be continued by incorporating the same filler in a fully biodegradable composite along with hydroxide-based fire retardant fillers.

## Supporting information

S1 Data Source data file. (XLSX)
